# Photobiomodulation Leads to Reduced Oxidative Stress in Rats Submitted to High-Intensity Resistive Exercise

**DOI:** 10.1155/2018/5763256

**Published:** 2018-02-13

**Authors:** Helenita Antonia de Oliveira, Ednei Luiz Antonio, Gisela Arsa, Eduardo Tadeu Santana, Flávio André Silva, Daniel Arruda Júnior, Simone dos Santos, Paulo de Tarso Camillo de Carvalho, Ernesto Cesar Pinto Leal-Junior, Amanda Araujo, Kátia De Angelis, Danilo Sales Bocalini, José Antonio Silva Junior, Paulo José Ferreira Tucci, Andrey Jorge Serra

**Affiliations:** ^1^Biophotonic Department, Universidade Nove de Julho, São Paulo, SP, Brazil; ^2^Cardiology Division, Escola Paulista de Medicina, Universidade Federal de São Paulo (UNIFESP), São Paulo, SP, Brazil; ^3^Postgraduate Program of Physical Education, Universidade Federal de Mato Grosso (UFMT), Cuiabá, MT, Brazil; ^4^Postgraduate Program in Nutrition, Food and Metabolism, Universidade Federal de Mato Grosso (UFMT), Cuiabá, MT, Brazil; ^5^Laboratory of Translational Physiology, Universidade Nove de Julho, São Paulo, SP, Brazil; ^6^Centro de Educação Física e Desporto, Universidade Federal do Espírito Santo (UFES), Vitória, ES, Brazil

## Abstract

The aim of this study was to determine whether oxidative stress markers are influenced by low-intensity laser therapy (LLLT) in rats subjected to a high-intensity resistive exercise session (RE). Female Wistar rats divided into three experimental groups (Ctr: control, 4J: LLLT, and RE) and subdivided based on the sampling times (instantly or 24 h postexercise) underwent irradiation with LLLT using three-point transcutaneous method on the hind legs, which was applied to the gastrocnemius muscle at the distal, medial, and proximal points. Laser (4J) or placebo (device off) were carried out 60 sec prior to RE that consisted of four climbs bearing the maximum load with a 2 min time interval between each climb. Lipoperoxidation levels and antioxidant capacity were obtained in muscle. Lipoperoxidation levels were increased (4-HNE and CL markers) instantly post-RE. LLLT prior to RE avoided the increase of the lipid peroxidation levels. Similar results were also notified for oxidation protein assays. The GPx and FRAP activities did not reduce instantly or 24 h after RE. SOD increased 24 h after RE, while CAT activity did not change with RE or LLLT. In conclusion, LLLT prior to RE reduced the oxidative stress markers, as well as, avoided reduction, and still increased the antioxidant capacity.

## 1. Introduction

Free radicals (FR) are molecules regularly produced by the body, leading to tissue injury or toxic compounds to tissues, and its accumulation results in tissue injury, DNA damage, and cellular dysfunction [1, 2]. The FR are responsible for damages caused by the oxidative stress and show accumulative effects that might contribute to the development of diseases such as diabetes, arteriosclerosis, and cancer [3, 4]. Physical exercise promotes the rise on the FR formation in function of the increment on the oxygen intake. Nevertheless, exercise training engenders adaptations able to unstiffen the damaging effects triggered by FR [5].

The FR are naturally formed in organisms through oxidative metabolic events, being extremely convenient as in conditions where the stimulation of the immunologic system is mandatory (e.g., macrophages utilize the hydrogen peroxide to destroy bacteria), in drug detoxification, and in the production of nitric oxide, an endothelium-derived relaxing factor imperative in processes that mediate the blood vessels relaxation [6, 7]. An excess of FR has been implied as both physiologically beneficial and pathologically harmful in the body. The FR has been demonstrated to act as intracellular signaling molecules for insulin signaling transduction in healthy tissues, and FR produced during exercise is also thought to play a key role in skeletal muscle adaptations associated with exercise training [8, 9]. In contrast to this physiologically beneficial FR, a chronic overproduction of FR systemically in skeletal muscle promotes oxidative stress and might contribute to the pathogenesis of type 2 diabetes [10], aging [11], and cancer cachexia [12].

Several damages can raise the free radical production, in which aerobic physical exercise shows to be a free radical trigger linked to mitochondrial pathway mainly as a result of increased oxygen consumption [13]. On the other hand, resistance exercise evokes temporary blood flow reduction between the concentric and eccentric stages of muscle followed by perfusion reestablishment [14]. This intermittent hypoxia/reperfusion is associated with ATP degradation, xanthine oxidase activation, and a higher production of oxygen-reactive species [15, 16]. In this regard, McBride et al. reported an increased plasmatic malondialdehyde level with 6 and 24 h after a single resistance exercise session [14]. Hudson et al. have shown higher levels of carbonylated protein at healthy men immediately and 60 min postresistance exercise [15]. Lastly, elevated levels of thiobarbituric acid reactive substances, glutathione, and products of protein oxidation were reported in healthy well-trained men with 10 min postresistance exercise [16].

An excessive increase of the oxidative stress during muscle work can cause damage to proteins and cellular dysfunction. Moreover, high-intensity exercises (e.g., upward of the second ventilatory threshold) are marked by decompensated metabolic acidosis and sarcolemma microinjury, which can lead to inflammation and a burst of free radicals [17, 18]. The implication of these findings is a delayed postexercise muscle recovery, hence, impairing the quality of training sessions [19]. This is a key issue mainly during intense training stages, in which the free radical burst can overcome the antioxidant chemical and enzymatic protection [20] to maintain redox homeostasis, inducing oxidative damages as observed by Rietjens et al. and Cakir-Atabek et al. after a single resistance exercise session in men [21, 22].

Several studies have reported beneficial effects of therapy with antioxidant agents, including tocopherol (vitamin E) and other antioxidants [13, 23]. There is a main interest for new strategies as well as for the low-intensity laser therapy (LLLT) [24]. The prior LLLT application to a run exercise was suitable to reduce oxidative stress and muscle damage in trained men [23]. It was shown that six weeks of LLLT treatment was appropriate to mitigate the increased concentration of thiobarbituric acid reactive substances in older rats [25]. In another study, a reduced content of carbonylated protein in the gastrocnemius muscle of the mdx mice with muscular dystrophy that underwent LLLT irradiations prior to a high-intensity exercise on a treadmill was reported [26]. To our knowledge, no existing studies have evaluated the repercussion of LLLT on the oxidative stress induced by a bout of resistance exercise. Therefore, this study was designed to determine if the levels of oxidative stress markers are influenced by LLLT in rats subjected to a high-intensity resistance exercise session.

## 2. Material and Methods

### 2.1. Animals and Experimental Design

**Figure 1:**
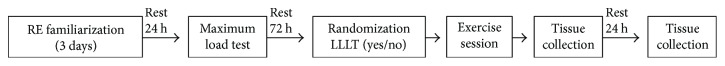
Timeline of experimental procedures.

Forty-eight female Wistar rats (*Rattus norvegicus* Albinus), weighing 200–250 g and aged 12 weeks, were obtained from the animal facility of the Federal University of São Paulo, São Paulo, SP, Brazil, and they were kept under controlled environments of light and temperature, with full water and food (Nuvilab CR-1; Global soluções para biotérios Inc., SP, BRA) access. Based on rats' availability in our animal's facility, several protocols were performed using female rats that have been published recently [27]. However, primarily, the same protocols were performed using both sexes and significant difference was not found between males and females in photobiomodulation experiments. Moreover, in a translational view, the landscape of female athletics has changed dramatically in the past three decades, in which female athletic participation in competitions has drastically increased worldwide [28]. The study protocol was approved by the Institutional Research Ethics Committee (8868250615), and experimental procedures were carried out in accordance with the guidelines stipulated by the Brazilian College of Animal Experimentation and the International Council for Laboratory Animal Science Standards. The rats were assigned for one of the three experimental groups, with some animals allocated per subgroup based on the sampling times (instantly or 24 h postexercise). Thus, three groups were as follows: *Ctr*, comprising rats that did not undergo resistance exercise or LLLT application; *RE*, comprising rats that only underwent a resistance exercise session. These rats were manipulated in the same way as those in the LLLT group, but the equipment was turned off; 4J, rats that were subjected to 4J LLLT prior to a resistance exercise session. Experimental timeline design is illustrated in Figure 1, in which the rats were initially adapted to the act of climbing for three consecutive days and following (post-24 h) the maximum load test. Then, the rats were kept at rest for 72 hours and randomized to undergo or not LLLT prior resistance exercise. Finally, the rats were euthanized for the removal of the gastrocnemius muscle instantly or 24 h postexercise.

### 2.2. Resistance Exercise Protocol

Rats were familiar to climb a ladder containing 54 vertical (slope: 80^o^) steps and a cage at the top as previously described by Sanches et al. [29]. The maximum load supported by the animal was determined as follows: (i) a load of 75% of body weight was attached to the rat's tail base; (ii) an additional load of 15% of the body weight was progressively attached to the tail in subsequent climbs until the animal failed to complete the climb to the top of the ladder. A 2 min rest was given between each climb attempt, and the load of the last complete climb was established as a maximum load. Overall, three climbs were required for the rats to reach maximum load. Resistance exercise session was carried out 72 h after maximum load test, in which this was defined based on a pilot study. It was observed that 72 h of rest between the maximal load test and the resistance exercise session results in the return of muscle injury markers (e.g., creatine kinase and lactic dehydrogenase) to the basal levels (data not shown). Resistance exercise session comprised four climbs bearing the maximum load with a 2 min time interval between each climb.

### 2.3. LLLT Protocol

A DMC Laser Photon III® model (São Carlos, SP, Brazil) was used as LLLT device. The application used the three-point transcutaneous method on the hind legs, in which the gastrocnemius muscle was irradiated at the distal, medial, and proximal points. Laser or placebo procedure (device off) was carried out 60 sec prior to resistance exercise. The Ctr group experienced the same experimental conditions that RE and 4J groups, but did not receive exercise or laser intervention. The laser device was fixed with the following parameters: wave length (830 nm); laser beam (0.028 cm^2^); output power (100 mW); power density (3.57 W/cm^2^); energy density (144J/cm^2^); total energy per point (4J); and irradiation time per point (40 s). The irradiation parameters were defined based on a previous study of our group (in per review analysis in the *Photochemistry and Photobiology* journal). It was observed that 4J irradiation attenuated muscle injury and inflammation in rats subjected to a resistance exercise session.

### 2.4. Physical Performance

The load, distance climbed, and time spent to complete each repetition were recorded to evaluate physical performance as previously described for rodents [30]. Thereby, the muscle work (MW) was calculated as follows:

- MW (joules) = mgh (“m,” comprising the external load fixed on the animal plus the body weight expressed in kg; “g,” acceleration due to gravity; “h,” climb distance expressed as meters). Results are expressed as mean ± standard error of mean to four climbs.
- Muscle power (MP) = J/s (“J,” represents the MW; “s,” time to perform the climbs). Results were obtained in milliwatts (mW) and presented for each climb.

### 2.5. Euthanasia and Tissue Sample

At the end of each experimental period (instantly and 24 h postexercise), the rats were identified, weighed, and then euthanized by intraperitoneal administration of urethane overdose (4.8 g kg^−1^, i.p.). Then, the gastrocnemius muscles of the right hind paw were quickly removed, rinsed in saline, and trimmed to remove fat tissue and visible connective tissue. The sample was stored at −80°C for further processing.

### 2.6. Lipid Peroxidation

The muscle was cut into small pieces and homogenized in an Ultra-Turrax blender with 1 g of tissue per 5 mL of buffer (150 mmol/L KCl; 20 nmol/L phosphate; pH 7.4). The homogenates were centrifuged at 600*g* for 10 min (−2°C), and protein concentration was assessed by the Lowry method. Lipid peroxidation was measured by the tert-butyl hydroperoxide-initiated chemiluminescence assay (CL), as it was previously described [31]. Moreover, 4-hydroxynonenal (4-HNE) expression was also used as a lipid peroxidation marker.

### 2.7. Western Blot

We followed the methods of Manchini et al. [32] and Melo et al. [33] for the muscle tissue processing and for protein extraction. Protein samples (20 *μ*g) were subjected to SDS-PAGE in 10–12% polyacrylamide gel. Separated proteins were transferred onto hydrophobic membranes (Hybond-P, Amersham Biosciences; Piscataway, NJ, USA), in which they were soaked in a blocking buffer (5% nonfat dry milk and 0.1% Tween 20 in PBS, pH 7.5) for 60 min at room temperature and then incubated overnight at 4°C with primary antibodies: rabbit anti-4-HNE (1 : 2000 dilution; Abcam, Cambridge, MA, USA); rabbit anti-SOD1 (1 : 5000 dilution; Abcam, Cambridge, MA, USA); rabbit anti-CAT (1 : 5000 dilution; Abcam, Cambridge, USA); goat anti-HSP70 (1 : 1000; Abcam, Cambridge, MA, USA); and anti-GAPDH (1 : 500; Santa Cruz Biotechnology, Santa Cruz, CA, USA). Then, membranes were washed five times and incubated for 60 min with horseradish peroxidase-conjugated goat anti-rabbit and rabbit anti-goat secondary antibodies (1 : 2000; Invitrogen, San Diego, CA, USA). Membranes were washed five times again with blocking buffer and then rinsed twice in PBS buffer. Bound antibody was detected by using chemiluminescence reagent for 60 s. The bands were imaged by using Amersham Imager 600 system (GE Health Care, Little Chalfont, UK, USA).

### 2.8. Oxidized Protein Assay

Measurement of carbonyl groups introduced into proteins by oxidative reactions was evaluated in gastrocnemius muscle with Western blot detection Abcam kit ab178020 (Abcam, Cambridge, MA, USA). An equal protein load (20 *μ*g) was used in all experimental groups, and protein carbonyl groups were measured according to the manufacturer's instructions.

### 2.9. Antioxidant Enzymes

Superoxide dismutase (SOD) activity was evaluated by the inhibition of the reaction between peroxide anion and pyrogallol, and catalase (CAT) activity was assessed by measuring the decrease in H_2_O_2_ absorbance at 240 nm [34]. Glutathione peroxidase (GPx) activity was determined by adding to the assay a mixture of 1 U/mL glutathione reductase and 2 mmol/L glutathione in 1 mL phosphate buffer. Homogenates were preincubated at 37°C for 30 minutes, NADPH and tert-butyl hydroperoxide were added, and the alteration in absorbance at 340 nm was recorded to calculate GPx activity [35].

### 2.10. Ferric Reduction Ability Power (FRAP)

The FRAP assay was applied in the analyses of the total antioxidant capacity of gastrocnemius muscle as adapted from elsewhere [36]. Homogenates tissue proteins were reacted with FRAP solution for 10 min (37°C), and sample absorbance was evaluated at 593 nm. Deionized water and Trolox (6-hydroxy-2,5,8-tetramethylchroman-2-carboxylic acid) were used as blank and standard, respectively. Absorbance readings at 593 nm were taken after 0.5 s and every 15 s thereafter during the monitoring period.

### 2.11. Statistical Analysis

Data were analyzed using GraphPad Prism software 5.0 (La Jolla, CA, USA). Kolmogorov-Smirnov and Levene tests were applied to verify normality and variance equality of data, respectively. Mann–Whitney test was carried in the analyses of physical performance. Two-way regular ANOVA and Bonferroni post hoc tests were applied to evaluate oxidative stress data. Kruskal-Wallis followed by Dunn's multiple comparison tests was applied to nonnormality data. Statistical significance was set at *p* ≤ 0.05. Data are expressed as mean ± standard error of the mean.

## 3. Results

**Figure 2:**
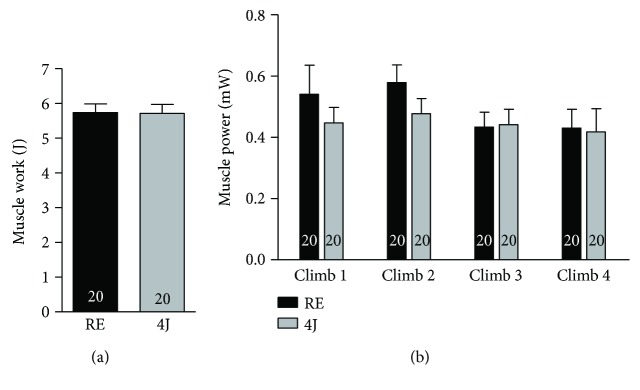
Physical performance data. (a) Muscle work developed by rats that only underwent a resistance exercise session (RE). (b) Muscle power developed by rats that were subjected to 4J LLLT irradiations prior to a resistance exercise session. Representative experiments are shown (*n* = 20 per group, RE and 4J).

A positive effect of LLLT was not identified on muscle performance. Thus, there were no significant differences among the experimental groups for total muscle work and muscle power in each climb session (Figure 2).

**Figure 3:**
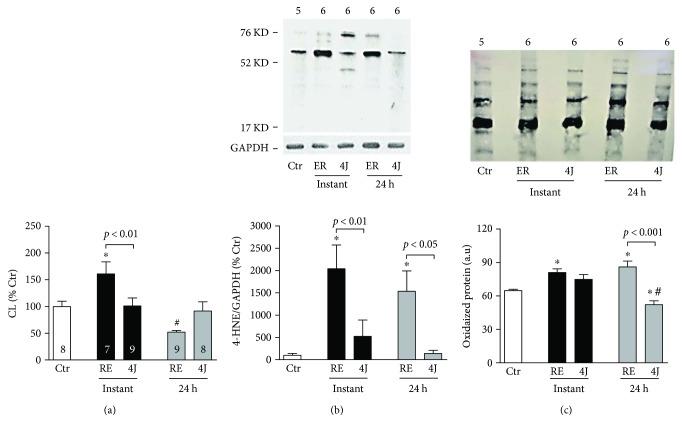
Lipid peroxidation levels in gastrocnemius muscle in control rats (Ctr), rats that only underwent a resistance exercise session (RE), and rats that were subjected to 4J LLLT irradiations prior to a resistance exercise session (4J). Data are representative of samples collected instantly and 24 h after exercise. (a) Chemiluminescence initiated by tert-butil. (b) 4-Hydroxynonenal (4-HNE) expression and (c) carbonyl protein were analyzed by Western blot (*n* = 4–6 per group). ^∗^*p* < 0.05 versus Ctr group; ^#^*p* < 0.05 versus instant time. Number of animals for each experiment is shown inside the bars and above the illustrations for (b) and (c).

Lipoperoxidation muscle parameters are shown in Figure 3. As evaluated by CL and 4-HNE expression, there was an elevated lipoperoxidation level post-RE. Both markers indicate a more evident lipoperoxidation increase instantly after exercise, in which LLLT resulted in similar peroxidation levels between 4J and Ctr groups. Exercised rats showed a significant increase in gastrocnemius muscle global protein carbonylation (for both time evaluation (Figure 3(c)). Exercise rats who were previously subjected to LLLT were protected from the increase in global protein carbonylation caused by RE, in which a more pronounced effect was observed 24 hours postexercise.

**Figure 4:**
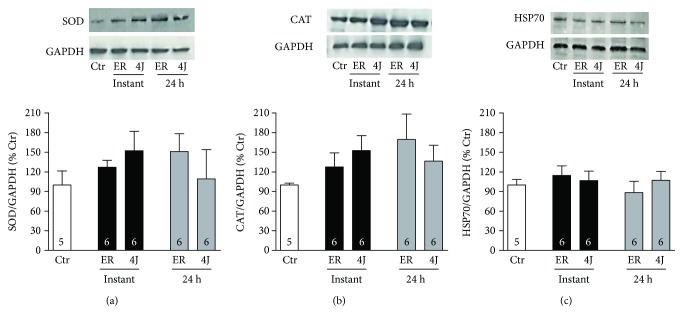
Protein expression of (a) superoxide dismutase (SOD), (b) catalase (CAT), and (c) 70 kilo Dalton heat shock protein (HSP70) in gastrocnemius muscle in control rats (Ctr), rats that only underwent a resistance exercise session (RE), and rats that were subjected to 4J LLLT irradiations prior to a resistance exercise session (4J). Data are representative of samples collected instantly and 24 h after exercise. Kruskal-Wallis and post hoc Dunn's tests were used for multiple comparisons. Number of animals for each experiment is shown inside the bars.

Regarding antioxidant enzymes, protein expression of SOD, CAT, and HSP70 was not affected by RE or LLLT at any time of analysis (Figure 4).

**Figure 5:**
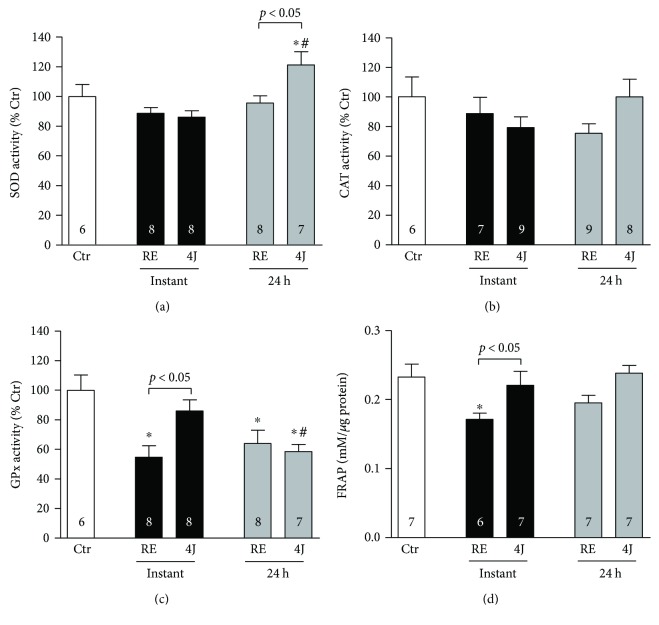
Enzymatic activity of (a) superoxide dismutase (SOD), (b) catalase (CAT), and (c) glutathione peroxidase (GPx) in gastrocnemius muscle in control rats (Ctr), rats that only underwent a resistance exercise session (RE), and rats that were submitted to 4J LLLT irradiations prior to a resistance exercise session (4J). (d) Total antioxidant capacity of gastrocnemius muscle by ferric reduction ability power assay (FRAP). Data are representative of samples collected instantly and 24 h after exercise. ANOVA two-way and Bonferroni post hoc tests were used for multiple comparisons. ^∗^*p* < 0.05 versus Ctr group; ^#^*p* < 0.05 versus instant time. Number of animals for each experiment is shown inside the bars.

On the other hand, a higher SOD activity 24 h postexercise has been reported in animals submitted to LLLT (Figure 5). There was no difference in CAT activity between conditions (Ctr versus RE versus 4J) or time (instant versus 24 h). However, a decrease in GPx activity was observed in the RE group compared with Ctr group, in which LLLT has normalized GPx activity in the 4J group to similar levels of the control group in analyses carried out instantly postexercise. In addition, the evaluation of FRAP was significantly increased instantly postexercise in the 4J group.

## 4. Discussion

There are studies involving animals and humans that show beneficial effects of LLLT on muscle damage and oxidative stress induced by aerobic exercise [23, 24, 26]. However, LLLT repercussion on oxidative stress evoked by RE is still unclear. Thus, our main findings were that oxidative stress and antioxidant capacity after a RE were modulated by the prior LLLT application. The LLLT reduced the oxidative stress and did not only avoid the reductions in antioxidant capacity but increased them.

A controversial issue is whether the LLLT can improve the physical performance. Leal Junior et al. have noticed a higher force peak in anterior tibial muscles of rats subjected to 1 and 3 J LLLT prior muscle fatigue linked to neuromuscular electrical stimulation [37]. On the other hand, Baroni et al. did not observe differences in eccentric peak torque in subjects submitted to LLLT before performing 75 maximal knee extensor eccentric contractions compared to the nonirradiated group [38]. Similarly, it was demonstrated by our results that rats irradiated with 4J energy did not show improved performance, as evaluated by muscle work and muscle power compared to nonirradiated rats.

Our resistance exercise protocol was suitable to increase oxidative stress as assessed by lipoperoxidation levels in muscle, corroborating previous data about resistance exercise [14–17, 22]. A decreased oxidative stress in muscles irradiated with 4J energy demonstrates to be of interest, in which more positive results were noticed for analyses of lipid peroxidation performed immediately after RE bout. Moreover, 4J rats exhibited protein oxidation levels similar to the Ctr group. These findings extend previous studies showing an antioxidant effect of LLLT when applied prior to aerobic exercise, as a progressive-intensity running protocol in humans and forced high-intensity treadmill in rats [23, 26]. An important aspect has been reported for our 24 postexercise findings. While the tert-butyl hydroperoxide-initiated chemiluminescence indicated lower lipid peroxidation in the nonirradiated exercised rats, the expression of 4-HNE indicated otherwise. The reasons for these findings are unclear, but may be associated with measurement methods. Some of these findings may be associated with a nonsingular stability of lipid hydroperoxides over time among experimental groups, which also makes it difficult to analyze lipid peroxidation. This chronicity of the process has relevant implications for measurement, in which hydroperoxides are unstable and extensive oxidation of a lipid can occur without a follow-up on the buildup in hydroperoxides [39]. Thus, changes in the mechanism of peroxide decomposition might change the amount generated without a lower lipid peroxidation rate in the RE rats as seen in the chemiluminescence assay. Therefore, we have applied two methods to confirm our findings of lipoperoxidation (i.e., CL and 4-HNE). This route corroborates that two or more different assay methods should be used to evaluate lipid peroxidation [40].

We have extended these analyses to determine whether a lower oxidative stress induced by the LLLT could also be associated with modulation of protein oxidation. Elevated cellular oxidants can result in posttranslational changes of proteins that can affect muscle function [41]. In fact, a high concentration of carbonyl derivates in gastrocnemius muscle was observed in the nonirradiated rats, mainly in later analyses (i.e., 24 h postexercise).

Reactive oxygen species (e.g., superoxide anions and hydroxyl radicals) cause the oxidation of membrane phospholipids, proteins, and DNA that has been implicated in several physiological disturbances. The adverse effects of an increase in free radicals can be countered by antioxidant enzymes, such as SOD, GPx, and CAT, and by nonenzymatic antioxidants [25]. In our case, antioxidant capacity instantly postexercise (i.e., GPx activity and FRAP) has been reduced in RE rats, but it was not observed when the LLLT was carried out. Moreover, there was an increased antioxidant SOD enzyme activity 24 h postexercise in 4J group when compared with the Ctr group. These results are in line with the findings of other studies showing that LLLT may modulate the antioxidant capacity [42, 43]. In this sense, the experimental results of the present study suggest that LLLT applied prior to resistance exercise session can increase the protection of the cells against oxidative stress induced by exercise. It could delay the impairments of muscle contractility that leads to fatigue [44] and hence contribute to the quality of training program. It should be noted that the CAT activity did not suffer any change with RE or LLLT. Our data agree with a study in which an acute exercise or LLLT [23] was not capable of modulating CAT activity. On the other hand, our data disagree that CAT activity may be reduced after a RE in women [45]. Therefore, the CAT activity affected by the RE remains controversial, and further studies should be performed.

Although the antioxidant enzyme activities have been changed by RE and LLLT, there were no significant changes in the protein expression. A null action of LLLT in the antioxidant protein expression is not a novelty. It has been demonstrated that LLLT does not alter SOD expression in subjects subjected to running [23]. Based on these findings, it may be proposed that LLLT modulate disruption of the physiological balance between the oxidant and antioxidant enzymes in exercised muscle, most likely by favoring increased enzymatic activity. In this context, the data from this study suggest that a comprehensive analysis of antioxidant enzymes should contain their expression as well as activity. Lastly, studies are showing that photobiomodulation can improve mitochondrial function and mitigate FR generated during exercise training [46]. Therefore, the mitochondrial function shows to be a key target, in which the role of photobiomodulation in altering the generation of FR induced by resistance exercise shows to be a goal to be investigated.

A major issue is that the increase in oxidative stress shows to be a common physiological process induced by resistance exercise. Regarding this, our LLLT-induced oxidative stress inhibition findings may fall into the debate whether antioxidant treatment could attenuate or eliminate exercise-induced adaptive response in skeletal muscle [47]. Although some researchers have not reported ablation of beneficial exercise role with an antioxidant treatment [48], it was demonstrated that giving antioxidant (e.g., vitamin C) the whole adaptive response was knocked out [49]. Consequently, the impact of the LLLT-induced oxidative stress prevention in important physiological process as exercise-induced adaptation (e.g., muscle hypertrophy) shows to be investigated.

In conclusion, LLLT prevented the increase of oxidative stress markers in the rats' gastrocnemius muscle subjected to a high-intensity resistance exercise session. The 4J energy irradiation was associated with improvement in the antioxidant defense linked to higher SOD and GPx activities (24 h and instantly postexercise, resp.). Moreover, a significant difference was found in muscle FRAP between RE and 4J rats instantly postexercise, indicating that a nonenzymatic total antioxidant capacity was preserved with LLLT. The ascorbic acid, α-tocopherol, proteins, and bilirubin are the main contributors to FRAP of plasma, detected by the FRAP assay [36, 50]. Thus, the data suggest that LLLT modifies the concentration of these antioxidant molecules in the exercise muscle. Overall, these results indicate that LLLT could be an important approach to counteract the supraphysiological production of reactive oxygen species during RE.
